# Peripheral neuropathy after extra-corporeal membrane oxygenation therapy in children: A case report

**DOI:** 10.1097/MD.0000000000027735

**Published:** 2021-11-12

**Authors:** Jun Young Ko, Mi rim Lee, Eun-Hye Ha, Aram Kim

**Affiliations:** a Department of Rehabilitation Medicine, Myongji Hospital, 14-55 Hwasu-ro, Deokyang-gu, Goyang-si, Gyeonggi-do, Republic of Korea; b Division of Pulmonary and Critical Care Medicine, Myongji Hospital, 14-55 Hwasu-ro, Deokyang-gu, Goyang-si, Gyeonggi-do, Republic of Korea.

**Keywords:** case report, extra-corporeal membrane oxygenation, pediatrics, peripheral nerve injuries

## Abstract

**Rationale:**

In recent years, the use of extracorporeal membrane oxygenation (ECMO) treatment for pediatric patients with cardiorespiratory failure has increased, with emphasis being given to the prevention of complications in ECMO-treated patients. Several studies have reported ECMO-related central nervous system complications, such as intracranial hemorrhage, cerebral infarction, and seizure. However, few cases of peripheral nerve injury have been reported in ECMO-treated adults; there have also been no reported cases of peripheral nerve injury in the pediatric population.

**Patient Concerns:**

Two pediatric patients aged 16 and 6 experienced motor weakness in the extremities after the insertion of ECMO equipment.

**Diagnoses:**

They were diagnosed with peripheral nerve injuries through an electrodiagnostic study that showed femoral/sciatic neuropathies and brachial plexopathy. Arteriography and doppler sonography was performed to find the cause of peripheral nerve injury, and this may be the results of vascular compromise and compressive injuries, respectively.

**Interventions:**

Surgical embolectomy was performed to remove thrombus in one patient. Two patients received orthosis, and physical therapy and occupational therapy were performed to prevent contracture and improve strength and functional use.

**Outcomes:**

Two pediatric patients showed a gradual improvement in motor power and function.

**Lessons:**

Through this case report, we present rare ECMO-related complications and emphasize the importance of early diagnosis and monitoring of peripheral nerve injury in ECMO-treated children.

## 1 Introduction

Extra-corporeal membrane oxygenation (ECMO) has remarkably progressed over the recent years and has become a therapeutic option in adults and children with cardiorespiratory failure.^[1,2]^ Neurological complications after ECMO occur frequently, with the prevalence and types of neurologic complications varying according to patient population.^[3]^ Based on data from the National Extracorporeal Life Support Organization (ELSO) Registry, neurological complications have been reported in 7.1% of ECMO-treated adults, with intracranial hemorrhage being the most common, followed by acute ischemic stroke in adults.^[4,5]^ An ELSO registry analysis of the pediatric population showed that intracranial hemorrhage, cerebral infarct, and seizures occurred in 7.4%, 5.7%, and 8.4% of all ECMO-treated children, respectively.^[6]^ Many institutions have protocols for central nervous system monitoring such as brain imaging and electroencephalography in ECMO-treated children, weekly cranial ultrasonography in neonates, and computed tomography (CT) or magnetic resonance imaging in older children with new neurological deficits. Compared to the well-described complications of the central nervous system after ECMO, few cases of peripheral nerve injury have been reported in adults.^[7–9]^ Survival rates are higher in children than in adults with cardiorespiratory failure,^[10]^ and peripheral nerve injury can contribute significantly to the quality of life of ECMO-surviving children. Here, we report 2 pediatric cases of ECMO-related peripheral nerve injury.

## 2 Ethics statement

Institutional review board approval was not necessary since this case report is not considered research. Written informed consent was obtained from the patient and parent for publication.

## 3 Case report

### 3.1 Case 1

**Figure 1:**
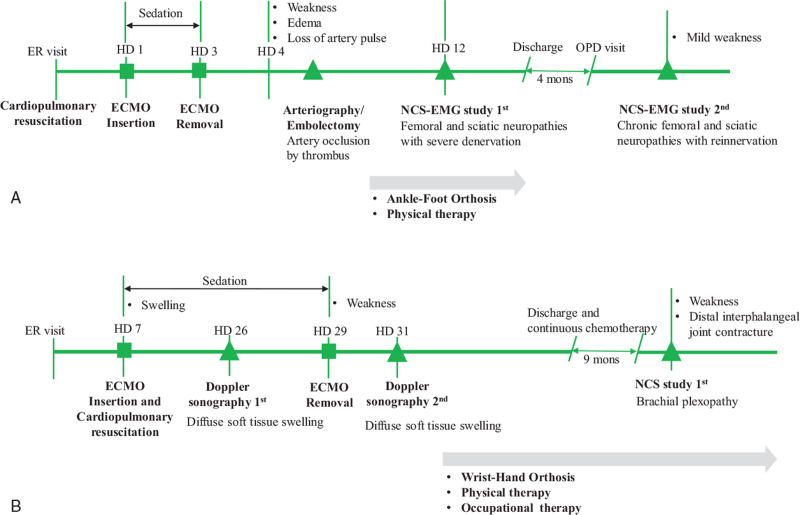
Timeline of the present cases: (A) Case 1, (B) Case 2. CPR = cardiopulmonary resuscitation, DIP joint = Distal interphalangeal joint, ECMO = extra-corporeal membrane oxygenation, EMG = electromyography, ER = emergency room, HD = hospital day, NCS = nerve conduction study, OPD = outpatient clinic.

A 16-year-old boy without vascular disease or other comorbidities was admitted to the hospital after taking herbal medicine containing *Aconiti ciliare tuber* extract. Refractory ventricular tachycardia occurred, and a total of 30 direct current shocks and 2 hours of chest compression were performed in the emergency room, resulting in successful resuscitation. On hospital day (HD) 1, venoarterial extra-corporeal membrane oxygenation (VA-ECMO) was placed with the right inferior vena cava and common femoral artery as a result of reduced cardiac function with an ejection fraction (EF) of 10% on portable transthoracic echocardiogram; 16.5F arterial cannula, 21F venous cannula, and ultrasound-guided intervention were performed. On HD 3, EF recovered to 40% on portable transthoracic echocardiogram, VA-ECMO was removed, and sedation was weaned off. On HD 4, the patient became alert and noted weakness and paresthesia in his right lower extremity, especially below the knee. Right leg edema and loss of the dorsalis pedis artery pulse were observed without soft tissue injury or hematoma at the ECMO catheter insertion site. Motor weakness was assessed using the Medical Research Council scale: hip flexor 4/5, knee extensor 4/5, ankle plantar flexor 4/5, and ankle dorsiflexor 1/5. Arteriography was performed to confirm right lower extremity artery patency; a thrombus was also observed, causing a total occlusion from the right external iliac artery to the common femoral artery. Distal flow did not recover despite 2 angioplasty trials; therefore, surgical embolectomy was performed. On HD 12, a nerve conduction study (NCS) was performed to assess nerve injury. The sensory NCS showed no response in the right superficial peroneal and sural nerves, and low amplitude in the right saphenous nerve. The motor NCS showed no response in the right common peroneal (including the tibialis anterior [TA]) and femoral nerves, and low amplitude in the right tibial nerve. Electromyography (EMG) showed abnormal spontaneous activities and reduced recruitment motor unit action potentials in the right vastus lateralis, TA, peroneus longus, medial gastrocnemius, and semimembranosus. These findings are consistent with right femoral and sciatic neuropathies with severe acute axonal degeneration (Table 1). On HD 15, he was referred to the rehabilitation department where an ankle-foot orthosis was attached and physical therapy (passive range of motion exercise, isometric exercise) was performed. After 4 months, a follow-up electrodiagnostic study was performed. The sensory NCS (right superficial peroneal and sural nerves) and motor NCS (right common peroneal, tibial, and femoral) showed improved amplitude compared to the previous study. EMG findings showed the disappearance of abnormal spontaneous activities in the right TA, peroneus longus, medial gastrocnemius, and semimembranosus muscles; however, large and polyphasic motor unit action potentials in the right vastus lateralis were still observed. This result was consistent with chronic femoral and sciatic neuropathies with reinnervation signs, indicating a good prognosis. Additionally, the patient showed improvement in muscle strength, not needing the ankle-foot orthosis anymore. We thought that the injury mechanisms in this case were femoral and sciatic neuropathies related to vascular compromise in the common femoral artery, which supplies the femoral and sciatic nerves via the femoral artery itself and the medial circumflex femoral artery branch, respectively. Figure 1 summarizes the timeline (Fig. 1A).

**Table 1:** Nerve conduction study and electromyography findings of case 1.

NCS summary table			
Nerve	Initial/follow-up	Initial/follow-up	Initial/follow-up
Motor	Latency (ms)	Amplitude (mV)	Velocity (m/s)
R peroneal (EDB recording)	NR^∗^/NR^∗^	NR^∗^/NR^∗^	NR^∗^/NR^∗^
L peroneal (EDB recording)	3.44/4.58	9.5/12.7	47.3/45.8
R peroneal (TA recording)	4.11/4.38	0.6^∗^/5.7	69.8/46.3
R tibial (AH recording)	4.53/4.06	16.0^∗^/30.5	42.4/44.9
L tibial (AH recording)	4.06/4.01	25.2/34.6	44.6/47.4
R femoral (VM recording)	NR^∗^/2.34	NR^∗^/6.2^∗^	
L femoral (VM recording)	6.09/2.66	7.6/27.1	
Sensory	Latency (ms)	Amplitude (μV)	Velocity (m/s)
R sural	NR^∗^/4.01	NR^∗^/2.0^∗^	NR^∗^/27.3^∗^
L sural	3.02/2.81	11.6/29.6	37.9/36.5
R superficial peroneal	NR^∗^/4.01	NR^∗^/1.3^∗^	NR^∗^/30.5^∗^
L superficial peroneal	3.13/3.23	14.0/14.7	42.7/43.7
R saphenous	3.18/3.96	2.1/1.6^∗^	36.8/24.8
L saphenous	3.13/3.23	3.4/5.3	37.9/36.2
EMG summary table			
Muscle	Fib and PSW initial/follow-up	MUAP initial/follow-up	Recruitment initial/follow-up
R vasatus lateralis	+/+	Normal/large and poly	Reduced/reduced
R tibialis anterior	+/–	Small/normal	Reduced/reduced
R peroneus longus	+/–	Small/normal	Reduced/reduced
R gastrocnemius	+/–	Small/normal	Reduced/reduced
R semimemtranosus	+/–	Small/normal	Reduced/reduced

AH = abductor halluces, EDB = extensor digitorum brevis, EMG = electromyography, Fib = fibrillation, L = left, MUAP = motor unit action potential, NCS = nerve conduction study, NR = no response, PSW = positive sharp wave, R = right, TA = tibialis anterior, VM = vastus medialis.

∗ Abnormal finding.

### 3.2 Case 2

A 6-year-old girl was diagnosed with pleuropulmonary blastoma and underwent video-assisted thoracic surgery right upper lobectomy 4 years prior. She recently noted a giant mass in the right hemithorax and started chemotherapy. Three months after recurrence, she was admitted to the pediatric department because of neutropenic fever. On HD 7, VA-ECMO was placed with the right internal jugular vein and common carotid artery as a result of heart failure progression with an EF of 20% due to septic shock and chemotherapy-induced cardiomyopathy; 12F arterial cannula, 17F venous cannula, Permanent Life Support pump, and Permanent Life Support Poly-methyl pentene oxygenator were used. Plasmapheresis extravasation occurred during ECMO insertion, and swelling of the right arm was noted. Muscle strength was not checked because she was in a state of deep sedation consciousness during ECMO treatment. On HD 26, Doppler sonography was performed due to continuous swelling of her upper extremity, and no prominent thrombus with intact blood flow and diffuse soft tissue swelling was observed in the right upper arm and forearm. On HD 29, heart failure improved and VA-ECMO was removed, and the patient was weaned off sedation. Motor strength was as follows on the Medical Research Council scale: shoulder flexor 2/5, elbow flexor 2/5, wrist flexor 1/5, and finger flexor 1/5. Sensation was decreased in the right upper extremity. On HD 31, Doppler sonography was repeated; diffuse soft tissue swelling was observed, similar to previous findings. She was referred to the rehabilitation department, where the patient received a wrist-hand orthosis; physical therapy and occupational therapy were also performed to maintain range of motion and functional use. Nine months after ECMO removal, followed by continuous chemotherapy for recurrent cancer lesions, NCS was performed to check for nerve injury of the right upper extremity. The sensory NCS showed no response in the right median, ulnar, superficial radial, medial antebrachial cutaneous, and lateral antebrachial cutaneous nerves. The motor NCS showed no response in the right median, ulnar, and radial nerves, and low amplitude in the right axillary nerve. Additionally, the right median and ulnar nerves showed no response to F-waves. EMG was not performed due to soft tissue swelling, and the reinnervation sign was not checked. According to the findings of this NCS only, right brachial panplexopathy involving the middle and lower trunk with severe axonotmesis and upper trunk with mild to moderate partial axonotmesis. The possible mechanism in this case is a local compressive injury from soft tissue swelling or previous hematoma formation on the brachial plexus. Other possible mechanisms of injury are direct nerve injury during intervention; internal jugular venous catheterization results in a higher incidence of plexus injury in cardiac surgery.^[11]^ Nine months after ECMO removal, motor strength in the right upper extremity, except the distal muscles, slightly improved; shoulder flexor 3/5, shoulder abductor 3/5, elbow flexor 4/5, elbow extensor 2/5, finger flexor 1/5, and finger extensor 1/5. However, distal interphalangeal joint contracture was observed because of continuous distal weakness and poor compliance with the brace. Figure 1 summarizes the timeline (Fig. 1B).

## 4 Discussion

ECMO is an effective life-saving treatment for patients with cardiorespiratory failure. Peripheral nerve injury with ECMO therapy is very uncommon compared to neuralgic complications of the central nervous system. We examined 2 children with peripheral nerve injuries after ECMO. In previous adult cases, local compressive injury from hematoma and acute limb compartment syndrome were suggested as mechanisms of neural injury.^[7–9]^ Other possible mechanisms include direct injury during the intervention, direct pressure from the catheter, and compromised blood supply to the nerve. In our 2 cases, peripheral nerves related to vascular compromise and compressive injury were the most probable mechanisms.

As neuralgic complications have a significant impact on the survival and quality of life in ECMO-treated children, early detection of peripheral neural injury and preventive strategies are required. There are several guidelines for neuromonitoring of the central nervous system, including neuroimaging (cranial ultrasound, CT, and MRI), electroencephalography, transcranial Doppler ultrasound, cerebral near-infrared spectroscopy, biomarkers (S100β, GFAP, neuron-specific enolase, and IL-6), somatosensory evoked potentials, and optic nerve sheath diameter.^[3]^ However, peripheral nerve injury has rarely been reported in previous cases, and there are no recommendations for neuromonitoring of the peripheral nervous system. A previous study recommended daily interruption of sedation and neuromuscular blocking agents for daily neurological assessments of the central nervous system.^[4]^ This proposed algorithm is appropriate for monitoring not only the central nervous system but also peripheral nerve injury, and we suggest weaning or titration from sedation and daily neurologic examination for monitoring. Any neurological deficit during ECMO therapy requires immediate evaluation. If there are no central nervous system complications, clinicians immediately need to proceed with peripheral nerve evaluation. Imaging evaluation (ultrasound, CT, and MRI) is recommended for the early detection of lesions, such as hematoma, acute limb compartment syndrome, and vascular compromise requiring emergent management.^[9]^ Subsequently, EMG and somatosensory evoked potentials are suggested to confirm the accurate localization of neural injury, prognosis, and rehabilitation plans. In our cases, the limitation of daily neurologic examination due to sedation may have delayed the early detection of peripheral nerve injury and appropriate intervention.

The risk factors of neurologic injury include underlying physiological conditions (prior cardiogenic shock or cardiac arrest), ECMO itself (cannula type), and inflammatory response.^[12]^ Several studies have reported the effectiveness and safety of venovenous extra-corporeal membrane oxygenation (VV-ECMO) based on comparable mortality and less common neurologic injury.^[13,14]^ They suggested that VV-ECMO has been favored over VA-ECMO in the pediatric population, except in several indications where VA-ECMO is preferred, such as inadequate cardiac function and lung transplantation bridge-ECMO.^[15–17]^ It seems likely that these are due to a more stable hemodynamic transition than arterial assessment and the low risk of embolization due to the filtering of thrombi by blood returning to the venous system in VV-ECMO.^[3,13]^ Additionally, due to the close anatomical proximity of the various important structures and the narrow vascular diameter in the pediatric population, ultrasound-guided procedures are recommended for invasive intervention (vascular access and regional anesthetic technique) in children for anatomical assessment and accurate needle insertion.^[18–20]^ Image-guided ECMO intervention with real-time ultrasound or fluoroscopy has to be suggested in neonatal intensive care unit and pediatric intensive care unit patients to decrease neurologic injury. Although the pathophysiology of complications after ECMO remains unclear, several previous studies have reported ECMO-induced inflammatory response effect complications.^[21]^ The exposure of blood to the non-endothelialized ECMO circuit activates the complement and contact system, leading to the induction of the coagulation pathway and production of pro-inflammatory mediators. This pathophysiology can contribute to complications of peripheral nerve injury. Further studies are required to focus on anticoagulation and novel anti-inflammatory therapy depending on the pathophysiology after higher quality studies in this context.

As survival after ECMO improves with advances in technology and patient care, neurologic complications have a significant impact on the quality of life after ECMO survival, especially in the pediatric population.^[6]^ However, peripheral nerve injury related to ECMO is underappreciated; for example, the ELSO Registry does not track peripheral nervous system complications, and the true incidence of peripheral nerve injury in adults and children remains unknown. Further data collection is needed to determine the prevalence of complications of the peripheral nervous system, including neuropathy, myopathy, and motor neuron disease. The protocol for monitoring and prevention of peripheral neural injury is required based on the characteristics and pathophysiology according to age.

## Author contributions

**Supervision:** Aram Kim.

**Visualization:** Mi rim Lee.

**Writing – original draft:** Jun Young Ko.

**Writing – review & editing:** Eun-Hye Ha, Aram Kim.
